# Tacrolimus‐induced hypomagnesemia and hypercalciuria requires FKBP12 suggesting a role for calcineurin

**DOI:** 10.14814/phy2.14316

**Published:** 2020-01-06

**Authors:** Brittany D. K. Gratreak, Elizabeth A. Swanson, Rebecca A. Lazelle, Sabina K. Jelen, Joost Hoenderop, René J. Bindels, Chao‐Ling Yang, David H. Ellison

**Affiliations:** ^1^ Division of Nephrology and Hypertension Department of Medicine Oregon Health and Science University Portland OR USA; ^2^ Radboud Institute for Molecular Life Sciences Radboud University Medical Center Nijmegen Netherlands; ^3^ Renal Section Veterans Affairs Portland Health Care System Portland OR USA

**Keywords:** calcineurin inhibitor, calcium, distal tubule, magnesium, tacrolimus

## Abstract

Calcineurin inhibitors (CNIs) are immunosuppressive drugs used to prevent graft rejection after organ transplant. Common side effects include renal magnesium wasting and hypomagnesemia, which may contribute to new‐onset diabetes mellitus, and hypercalciuria, which may contribute to post‐transplant osteoporosis. Previous work suggested that CNIs reduce the abundance of key divalent cation transport proteins, expressed along the distal convoluted tubule, causing renal magnesium and calcium wasting. It has not been clear, however, whether these effects are specific for the distal convoluted tubule, and whether these represent off‐target toxic drug effects, or result from inhibition of calcineurin. The CNI tacrolimus can inhibit calcineurin only when it binds with the immunophilin, FKBP12; we previously generated mice in which FKBP12 could be deleted along the nephron, to test whether calcineurin inhibition is involved, these mice are normal at baseline. Here, we confirmed that tacrolimus‐treated control mice developed hypomagnesemia and urinary calcium wasting, with decreased protein and mRNA abundance of key magnesium and calcium transport proteins (NCX‐1 and Calbindin‐D_28k_). However, qPCR also showed decreased mRNA expression of NCX‐1 and Calbindin‐D_28k_, and TRPM6. In contrast, KS‐FKBP12^−/−^ mice treated with tacrolimus were completely protected from these effects. These results indicate that tacrolimus affects calcium and magnesium transport along the distal convoluted tubule and strongly suggests that inhibition of the phosphatase, calcineurin, is directly involved.

## Graphical abstract

We showed that deletion of the tacrolimus‐binding protein, necessary for tacrolimus to inhibit calcineurin, completely prevented the effects on divalent ion metabolism. This was associated with protection of transport proteins in the distal convoluted tubule.

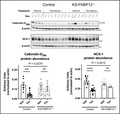

## 1 INTRODUCTION

Adverse effects of immunosuppressive calcineurin inhibitors (CNIs) include renal magnesium wasting and hypomagnesemia, which may contribute to the development of new‐onset diabetes mellitus after kidney transplantation (NODAT) (Huang, Famure, Li, & Kim, 2016), and hypercalciuria, which may contribute to post‐transplant osteoporosis (Yu et al., 2014). Magnesium is reabsorbed primarily along the thick ascending limb (TAL) and distal convoluted tubule (DCT), whereas calcium is reabsorbed along the proximal tubule, as well as the TAL and DCT. Along the TAL, divalent cation reabsorption is largely paracellular, medicated by a specific group of claudins, which are regulated physiologically (Gong & Hou, 2017). In contrast, along the DCT, calcium and magnesium are reabsorbed transcellularly. Both cations are reabsorbed across the apical membrane by members of the transient receptor potential (TRP) channels, TRPV5 for calcium and TRPM6 for magnesium (Lambers et al., 2006; Schaffers, Hoenderop, Bindels, & Baaij, 2018); the transcellular calcium transport pathway is now well established, and includes both apical and basolateral transporters, together with the intracellular calcium‐binding protein, calbinin‐D_28K_ (Lambers et al., 2006). Previous work has established that CNIs, such as tacrolimus, reduce the abundance of key magnesium and calcium transport and binding proteins. Tacrolimus treatment of rats reduced the abundance of both the distal tubule calcium channel TRPV5 and the calcium‐binding protein, calbindin‐D_28K_, at both the message and protein level (Lee et al., 2011; Nijenhuis, Hoenderop, & Bindels, 2004). Although these effects were proposed to contribute to hypercalciuria, direct effects on bone turnover were also suggested to contribute (Lee, Huynh, Lai, & Lien, 2002). With respect to magnesium wasting, which is recognized more commonly, tacrolimus reduced the abundance of TRPM6, the putative magnesium channel of the distal convoluted tubule (Nijenhuis et al., 2004). These effects on tubule calcium and magnesium transport, supplement abundant data showing that CNIs alter NaCl and K transport along the distal part of the nephron (Blankenstein et al., 2017; Hoorn et al., 2011). Together, these results suggest that CNIs are tubule toxins with segment‐specific effects on transport proteins along the nephron.

Tacrolimus must bind to an immunophilin, FKBP12, to inhibit the phosphatase calcineurin (also called protein phosphatase 3) and impart its immunosuppressive effects. Thus, tacrolimus could alter divalent cation homeostasis by inhibiting calcineurin or through ‘off target’ direct effects, independent of calcineurin. This distinction has potential therapeutic implications, as modulation of calcineurin activity via more specific inhibitors might avoid these effects. In fact, tubule toxicity may contribute to the other dreaded complication of CNIs, chronic kidney disease.

Deleting calcineurin in the kidney has led to confusing results, as there are several isoforms and different subunits. To clarify the role of calcineurin in tacrolimus‐induced cation transport inhibition, we adapted a model of tacrolimus toxicity that we developed previously (Hoorn et al., 2011; Lazelle et al., 2016) for use with mice lacking the tacrolimus‐binding protein, FKBP12. These mice appear normal, at baseline, indicating that FKBP12 itself is not necessary for normal calcium and magnesium homeostasis. We reasoned that mice lacking FKBP12 would be protected from effects of tacrolimus that require calcineurin inhibition. The results suggest that tacrolimus affects key divalent cation transport proteins in a segment‐specific manner, but that mice lacking FKBP12 are strikingly protected from these effects. The specificity and degree of protection conferred by FKBP12 deletion suggest strongly that protein phosphatase 3 plays a key role in modulating divalent cation transport along the distal part of the nephron.

## 2 METHODS

### 2.1 Animals

This study was approved by the Oregon Health and Science University Animal Care and Use Committee (Protocol #IP00000286) and adhered to the National Institutes of Health Guide for the Care and Use of Laboratory Animals. Laboratory mice (*Mus musculus*) were housed in a pathogen free facility and maintained on a 12‐hr:12‐hr light:dark cycle with free access to food and water. KS‐FKBP12^−/−^ mice were generated using the Pax8‐rtTA/TRE‐LC1 system as described (Lazelle et al., 2016).

To induce Cre‐mediated recombination, mice at 4–7 weeks of age were treated with 2 mg/ml doxycycline hyclate with 5% (w/v) sucrose in drinking water for two weeks to produce the KS‐FKBP12^−/−^ knockout genotype. Genetically identical age‐matched littermates were treated with 5% (w/v) sucrose in drinking water for two weeks to preserve the FKBP12^fl^/^fl^ control genotype. Male mice at 10–20 weeks of age were used for determination of plasma and urinary electrolytes and gene expression. Male and female mice at 10–13 weeks of age were used for determination of calbindin‐D_28K_ and NCX1 protein abundance.

### 2.2 Tacrolimus

Powdered tacrolimus (Cayman Chemical Company Catalog #10007965) was dissolved in a 3:1 solution of DMSO:TWEEN20 to 30 mg/ml. This solution was further diluted with PBS to 15 µg/ml. Mice received daily subcutaneous injections of tacrolimus (3 mg/kg body weight) or vehicle (3:1 DMSO:TWEEN20) for 18 days.

### 2.3 Metabolic cages

Mice were housed individually in metabolic cages (Hatteras Instruments). Mice received ad libitum water and gel diet, (Tekland TD.90228 sodium‐deficient diet with sodium adjusted to 0.49% (w/v) NaCl). Following a 2‐day acclimation period, urine was collected for 24 hr under water‐saturated mineral oil. Urine calcium concentration was measured using a cresolphthalein complexone colorimetric assay (Pointe Scientific #C7503‐120) with absorbance measured at 570 nm using a BioTek Synergy HT plate reader.

### 2.4 Plasma electrolytes

Under isoflurane anesthesia, whole blood was collected by cardiac puncture in heparinized tubes. A quantity of 100 µl was used to measure ionized calcium (chem 8 + cartridge, Abbott Point of Care). The remaining whole blood was centrifuged at 2,000*g* for 5 min at room temperature. The plasma was removed and stored at −80°C. Plasma magnesium was measured using colorimetric assay (Xylidyl blue assay, Pointe Scientific #HM929‐120) and absorbance measured at 530 nm using a BioTek Synergy HT plate reader.

### 2.5 qPCR

Kidneys were preserved at the time of collection in RNAlater, snap‐frozen in liquid nitrogen, and stored at 80°C. Total RNA was isolated from the kidneys with TRIzol reagent and treated with DNase to prevent genomic DNA contamination. cDNA was generated by reverse transcription of 1.5 µg RNA using M‐MLV reverse transcriptase. qPCR was performed using the Bio‐Rad iQTM SYBR^®^ Green Supermix kit according to the manufacturer's instructions. Gene expression was quantified using the Livak method (Livak & Schmittgen, 2001).

Following primers were used:

**Table:** 

TRPV5	F:5′ CTGGAGCTTGTGGTTTCCTC 3′
	R:5′ TCCACTTCAGGCTCACCAG 3′
TRPM6	F:5′ CTTACGGGTTGAACACCACCA 3′
	R:5′ TTGCAGAACCACAGAGCCTCTA 3′
NCC	F:5′ CTTCGGCCACTGGCATTCTG 3′
	R:5′ GATGGCAAGGTAGGAGATGG 3′
CLDN16	F:5′ GTTGCAGGGACCACATTAC 3′
	R:5′ GAGGAGCGTTCGACGTAAAC 3′
CLDN19	F:5′ GGTTCCTTTCTCTGCTGCAC 3′
	R:5′ CGGGCAACTTAACAACAGG 3′
NCX1.3	F:5′ CTCCCTTGTGCTTGAGGAAC 3′
	R:5′ CAGTGGCTGCTTGTCATCAT 3′
Calbindin‐D_28K_	F:5′ GACGGAAGTGGTTACCTGGA 3′
	R:5′ ATTTCCGGTGATAGCTCCAA 3′
GAPDH	F:5′ TAACATCAAATGGGGTGAGG 3′
	R:5′ GGTTCACACCCATCACAAAC 3′

### 2.6 Immunoblotting

Kidneys were removed, snap‐frozen in liquid nitrogen, and homogenized in chilled lysis buffer as described (McCormick et al., 2011). Half‐kidneys were homogenized and centrifuged at 3,500*g* for 15 min at 4°C. Total protein quantification was established using a colorimetric assay (Bio‐Rad DC Protein Assay) and 40 µg protein per sample was separated on a 4%–15% precast gel (Bio‐Rad Criterion Stain‐Free) before being transferred to a 0.45 µm PVDF membrane (Immobilon‐P) overnight at 150 mA at 4°C. The membrane was then blocked using nonfat milk protein in PBS with 0.1% (w/v) TWEEN20 for 1 hr at room temperature before being incubated overnight with primary antibody at 4°C. Antibody binding was detected using an HRP‐conjugated secondary antibody and visualized using Western Lightning Plus ECL. Prior to transfer, the gel was imaged using the PXi4 gel imaging system (Syngene). Total protein for each lane was measured using GeneTools software (Syngene). Membranes were imaged using the PXi4 gel imaging system and bands were quantified using GeneTools software. Bands were normalized to total beta‐actin protein.

### 2.7 Statistical analyses

Analyses were performed by two‐way ANOVA followed by Tukey′s multiple comparison procedure. For all analyses, *p* < .05 was considered statistically significant.

## 3 RESULTS

### 3.1 Deletion of renal FKBP12 prevents development of tacrolimus‐induced hypomagnesemia and hypercalciuria

**Figure 1:**
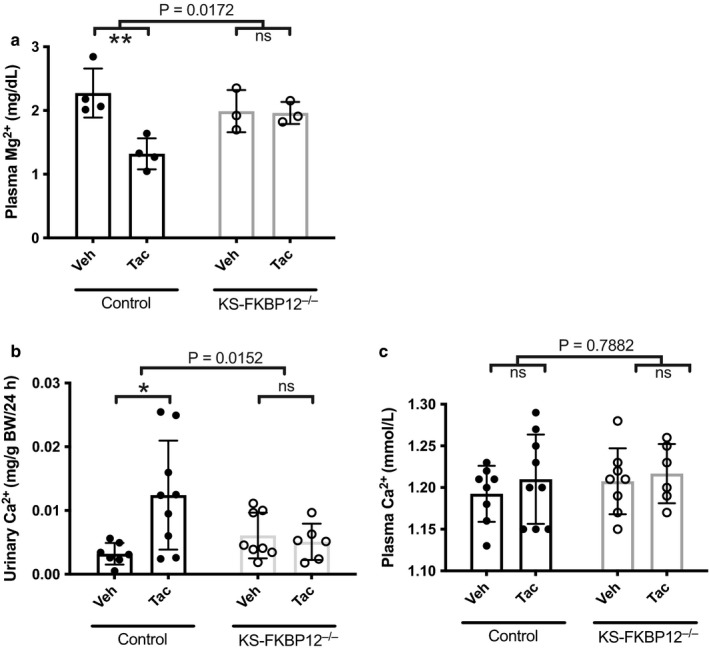
KS‐FKBP12^−/−^ mice are protected against tacrolimus‐induced hypomagnesemia and hypercalciuria. (a) Plasma [mg2+] in control and KS‐FKBP12^−/−^ mice treated with vehicle (Veh) or tacrolimus (Tac, *n* = 3–4 per group). (b and c) Urinary calcium excretion and plasma [Ca^2+^] in control and KS‐FKBP12^−/−^ mice treated with vehicle or tacrolimus (*n* = 6–9 per group). Statistical comparison with two‐way ANOVA followed by Tukey multiple comparison procedure. Exact p values indicate the significance of the interaction between treatment and strain. Additionally, tacrolimus reduced plasma [mg2+] and increased urinary Ca2^+^ in control mice, but not in KS‐FKBP12^−/−^ mice. *p < .05, **p < .01

In control (FKBP12^fl/fl^) mice, the plasma magnesium concentration was significantly lower in mice treated with tacrolimus than in mice treated with vehicle (Figure 1a). In contrast, tacrolimus treatment of KS‐FKBP12^−/−^ knockout mice did not reduce plasma magnesium concentration (Figure 1a). Thus, the effect of tacrolimus on plasma magnesium differed according to strain (2‐way ANOVA, interaction *p* = .0172). Although tacrolimus treatment did not alter plasma calcium concentration (Figure 1b), tacrolimus increased urinary calcium excretion significantly, compared with vehicle (Figure 1c). This effect was completely absent in the KS‐FKBP12^−/−^ mice treated with tacrolimus (2‐way ANOVA, interaction *p* = .0152).

### 3.2 Calcineurin inhibition via tacrolimus alters expression of key transport genes in the distal convoluted tubule (DCT)

**Figure 2:**
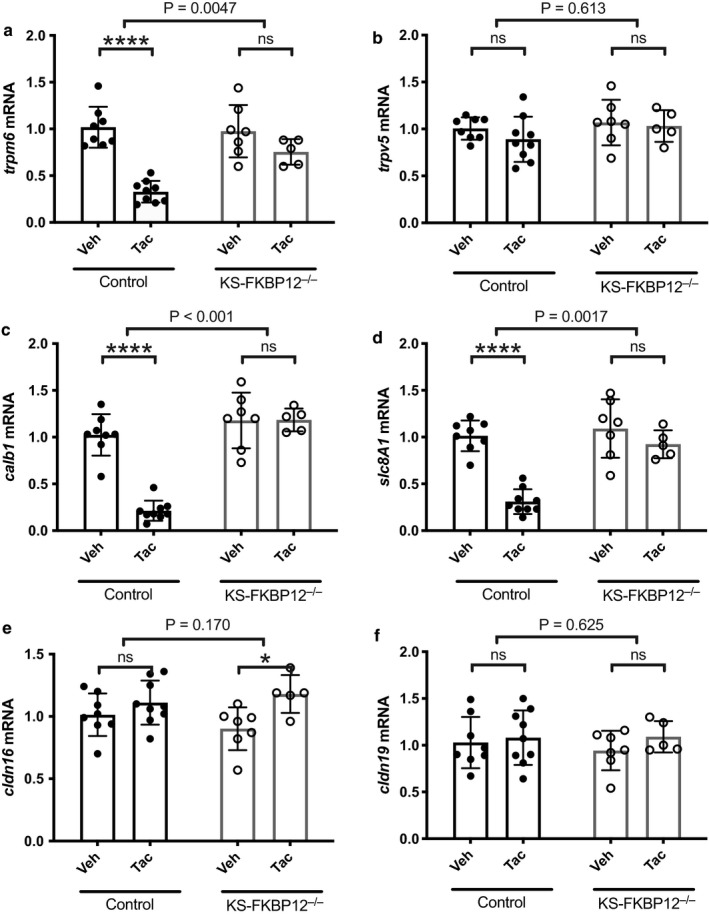
Effect of tacrolimus treatment on mRNA expression of transport proteins in control and KS‐FKBP12^−/−^ mice. (a–f) results of quantitative PCR of total RNA isolated from whole kidney harvested from control and KS‐FKBP12^−/−^ mice treated with vehicle or tacrolimus (*n* = 5–9 per group). Gene expression was quantified using the Livak method (Livak & Schmittgen, 2001). Statistical comparison with two‐way ANOVA followed by Tukey multiple comparison procedure. Exact p values indicate the significant of the interaction between treatment and strain. Additionally, there was a significant effect of tacrolimus treatment on TRPM6, calbindin‐D_28K_, and NCX‐1 mRNA abundance, but only in control mice. *p < .05, ****p < .0001

To determine the renal mechanisms by which tacrolimus alters divalent cation metabolism, we examined the effects of tacrolimus treatment on molecules that mediate magnesium and calcium transport along the DCT. Tacrolimus treatment of control mice decreased the abundance of mRNA encoding the magnesium channel, TRPM6, compared with vehicle (Figure 2a), whereas treatment of KS‐FKBP12^−/−^ had no effect on TRPM6. Similarly, tacrolimus treatment also decreased the abundance of mRNA encoding the calcium‐binding protein, calbindin‐D_28K_ (*calb1*), and the Na/Ca exchanger (*ncx1*), compared with vehicle (Figure 2b–d). In contrast, the same treatment did not affect the abundance of mRNA encoding these same genes when administered to KS‐FKBP12^−/−^. *Trpv5* mRNA abundance was similar in all groups regardless of genotype or treatment.

### 3.3 Tacrolimus treatment has no effect on key claudins expressed along the thick ascending limb

Unlike the DCT, where divalent cation transport is primarily transcellular, along the thick ascending limb, divalent cations traverse paracellular channels comprising claudins. Tacrolimus treatment did not affect mRNA encoding either Claudin 16 or Claudin 19 in either controls or KS‐FKBP12^−/−^ mice (Figure 2e and f).

### 3.4 FKBP12 deletion protects mice from tacrolimus‐induced decreases in Calbindin‐D_28K_ and NCX1 protein abundance

**Figure 3:**
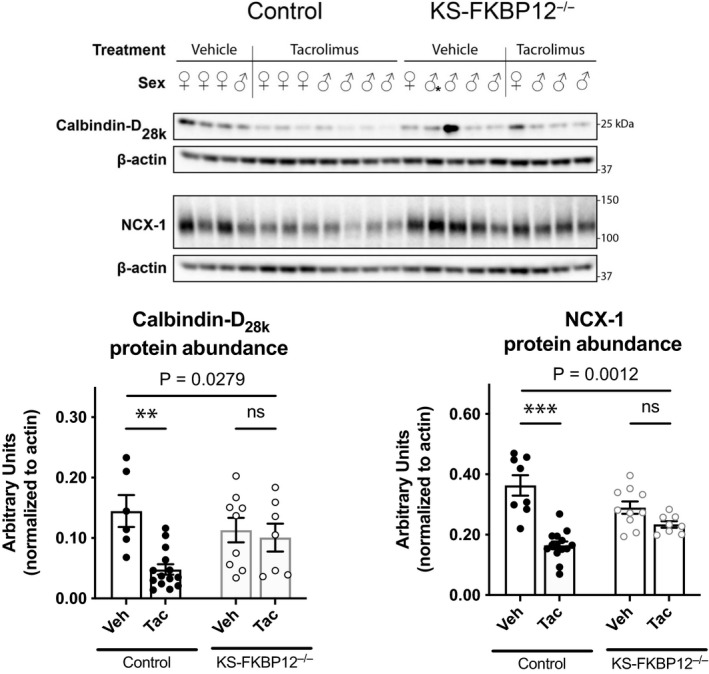
Effect of tacrolimus treatment on calbindin‐D_28K_ and Na/Ca exchanger protein abundance. (Top panel) Western blot of NCX‐1 and calbindin‐D_28K_ in KS‐FKBP12^−/−^ or control mice treated with vehicle or tacrolimus. (Bottom left) Quantification of calbindin‐D_28K_ protein. (Bottom right) Quantification of NCX‐1 protein. Statistical comparison with two‐way ANOVA followed by Tukey multiple comparison procedure. Exact *p* values indicate the significance of the interaction between treatment and strain. **p < .01, ***p < .001

To determine whether the effects observed on mRNA abundance resulted in similar changes in protein abundance, western blots of kidney tissue were performed. In control mice, the abundance of Calbindin‐D_28K_ was significantly lower in tacrolimus‐treated mice than in vehicle‐treated mice; this effect was absent in KS‐FKBP12^−/−^ mice (Figure 3a). FKBP12 deletion also protected mice from the effects of tacrolimus on NCX1 (Figure 3b).

**Figure 4:**
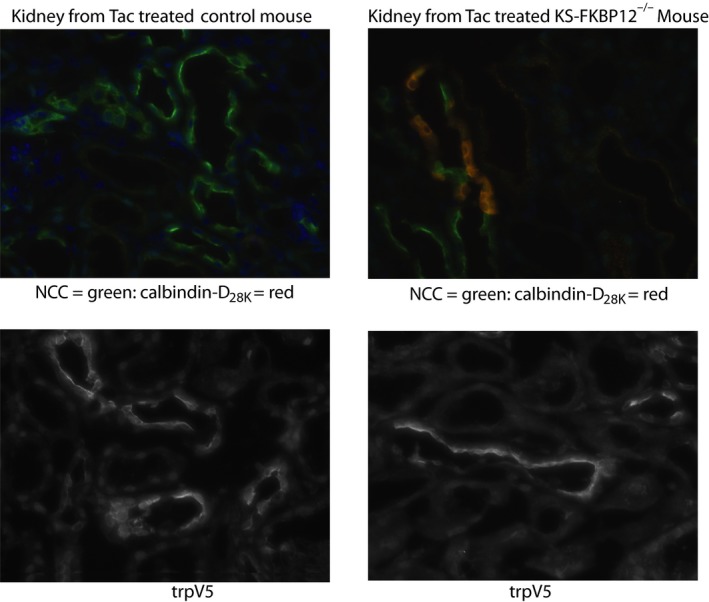
Immunofluorescent comparison of tacrolimus effects on control and KS‐FKBP12^−/−^ kidneys. The top panels are representative images showing labeling of NCC (green) and calbindin‐D_28K_. Note that calbindin‐D_28K_ is nearly absent in control kidneys following treatment with tacrolimus, but remains present in the knockout mice. The bottom panels are representative images showing labeling of TRPV5. Note that trpv5 remains clearly visible in tacrolimus‐treated control kidneys

We attempted to blot for trpv5, but the results were inconsistent. Tacrolimus has been reported to reduce trpv5 message and protein abundance (Lee et al., 2011; Nijenhuis et al., 2004), and yet we could not detect an effect at the message level. As western blots were inconsistent, we turned to immunofluorescence to estimate protein abundance. In contrast to the clear effect of tacrolimus to reduce Calbindin‐D_28K_ abundance, an effect blocked by FKBP12 deletion, the abundance of trpv5 appeared to be completely preserved following tacrolimus treatment, in both groups of mice (Figure 4). While these results are not quantitative, they do suggest that trpv5 abundance is relatively preserved during tacrolimus treatment.

## 4 DISCUSSION

Disturbed divalent cation metabolism is common following solid organ transplantation. Hypomagnesemia is a well‐recognized and common complication of CNI treatment (Lameris, Monnens, Bindels, & Hoenderop, 2012), and has been associated with the subsequent development of new onset diabetes after transplantation (Huang et al., 2016). Previous work suggested that renal losses of both magnesium and calcium are the major contributors to these side effects and that the effects reflect renal toxicity. Treatment with two chemically distinct CNIs, cyclosporine or tacrolimus, was found to reduce the abundance of calbindin‐D_28K_, an effect postulated to account for calcium wasting (Aicher et al., 1997; Yang et al., 1998). Nijenhuis and colleagues found that tacrolimus treatment increased fractional magnesium and calcium excretion and reduced expression of TRPV5 and calbindin‐D_28K_. They also showed that the transcript for TRPM6, a primary magnesium channel of the DCT was reduced by tacrolimus treatment (Nijenhuis et al., 2004). These actions were suggested to account for the hypomagnesemia and hypercalciuria that result from tacrolimus treatment, but several questions remained, as suggested by Naesens and colleagues (Naesens, Kuypers, & Sarwal, 2009). First, are these toxic effects of tacrolimus specific for the distal tubule, or do they reflect a more general toxic effect that includes other divalent cation transporting segments (such as the TAL). Second, are these effects a consequence of calcineurin inhibition, or are they toxic off‐target effects. The second question has substantial therapeutic implications, as distinct mechanisms would suggest that more specific approaches to calcineurin inhibition might be immunosuppressive, with less toxicity.

As mouse models attempting to delete calcineurin itself have proved complicated, in part because there are multiple isoforms and multiple subunits of calcineurin (Gooch, Guler, Barnes, & Toro, 2006; Gooch, Roberts, Cobbs, & Tumlin, 2007; Gooch, Toro, Guler, & Barnes, 2004), we previously developed a mouse model to test the role of calcineurin inhibition by tacrolimus (Lazelle et al., 2016). Tacrolimus must bind to a specific immunophilin, FKBP12, to inhibit calcineurin, and it is the combined tacrolimus–FKBP12 complex that binds to, and inhibits calcineurin; the dependency on FKBP12 is well established and supported by a vast literature (Nghiem, Pearson, & Langley, 2002). Yet, FKBP12 alone also has pleiotropic effects independent of tacrolimus and its constitutive deletion is embryonic lethal (Shou et al., 1998). To allow us to disrupt the effects of tacrolimus on calcineurin only in kidney tubules, we used the *Pax8‐rtTA* system, which generates an inducible model with target genes deleted primarily along kidney tubules. We previously validated the fidelity of this approach (Lazelle et al., 2016). We also showed that deletion of FKBP12 along kidney tubules of adult mice had no effect on calcium or magnesium balance, indicating that FKBP12 alone does not regulate divalent cation metabolism. Here, we tested whether deletion of FKBP12 in adult mice, which prevents the ability of tacrolimus to inhibit calcineurin would alter the functional effects of tacrolimus and its effects on calcium and magnesium transport proteins.

The results of tacrolimus treatment of control mice were largely consistent with prior work. We confirmed that treatment led to substantial reductions in the magnesium channel, TRPM6 and that several calcium transporting proteins (including the calcium chelator, calbindin‐D_28K_) were also reduced substantially, both at the message and protein level. Difficulty with TRPM6 antibodies precluded an accurate assessment of its abundance at the protein level, but this, too, has been reported as decreased previously (Nijenhuis et al., 2004). The only exception was with regard to TRPV5; although there was a hint of decrease in the tacrolimus‐treated group, as detected by others, it was not significant. To provide additional data regarding the effect of tacrolimus on TRPV5, we performed immunofluorescence, which also showed trpv5 presence following tacrolimus treatment. Thus, our model of tacrolimus toxicity led to robust effects at both the metabolic and molecular level.

We did not find any effects of tacrolimus treatment on either of two claudins that play key roles in divalent cation reabsorption along the TAL. In a cultured TAL model, cyclosporine was found to reduce claudin 16 abundance (Chang, Hung, Tian, Yang, & Wu, 2007), but in vivo treatment did not (Lee et al., 2011). Gong and You examined claudin‐14, a calcium‐regulatory claudin of the TAL. They noted that cyclosporine did not affect the abundance of claudin‐14, at baseline, but abrogated regulation by the calcium‐sensing receptor; this potential effect is not contradicted by the current studies. Thus, the current data largely support prior work suggesting that tacrolimus toxicity largely targets the distal parts of the nephron. As we could not accurately assess claudins at the protein level (we could not obtain reliable western blots), it remains possible that an additional defect along the thick ascending limb contributes to the effect of tacrolimus.

The results of FKBP12 deletion were striking and complete. At both the metabolic and at the molecular level, renal tubule FKBP12 deletion provided complete protection from tacrolimus toxicity. It remains possible that the tacrolimus–FKBP12 complex binds to a noncalcineurin protein and mediates these effects, but the fact that the effects of tacrolimus are a class effect, shared with chemically distinct CNIs (like cyclosporine), and provides strong support for the hypothesis that calcineurin itself plays a central role in modulating divalent cation transport proteins along the distal tubule. Interestingly, tacrolimus toxicity along the DCT appears specific for these pathways, as both NCC and NKCC2 can be activated by the same treatment (Blankenstein et al., 2017; Hoorn et al., 2011). In fact, it is not surprising that a protein phosphatase that is activated by intracellular calcium, calcineurin, would be involved in regulating key steps in divalent cation transport. Thus, the current results suggest that calcineurin plays a key role in regulating divalent ion transport along the distal convoluted tubule of the mammalian kidney.

## CONFLICT OF INTEREST

There are no conflicts of interest to report.

## AUTHOR CONTRIBUTIONS

D.H.E and C.L.Y conceived this study and analyzed data. B.D.K. G, E. A. S, R.A. L, S. K. J and J. H performed experiments. B.D.K.G, E.A.S, R.A.L and D.H.E prepared the figures and drafted manuscript. C.L.Y and D.H.E edited manuscript. D.H.E approved the manuscript.
